# Secondhand smoke is associated with peptic ulcer disease and gastroesophageal reflux disease in non-smokers in a large Taiwanese population study

**DOI:** 10.3389/fpubh.2024.1450481

**Published:** 2024-10-07

**Authors:** Pei-Chi Yen, Jiun-Hung Geng, Pei-Yu Wu, Jiun-Chi Huang, Huang-Ming Hu, Chao-Hung Kuo, Szu-Chia Chen

**Affiliations:** ^1^Department of Internal Medicine, Kaohsiung Medical University Hospital, Kaohsiung Medical University, Kaohsiung, Taiwan; ^2^Department of Urology, Kaohsiung Municipal Siaogang Hospital, Kaohsiung Medical University, Kaohsiung, Taiwan; ^3^Department of Urology, Kaohsiung Medical University Hospital, Kaohsiung Medical University, Kaohsiung, Taiwan; ^4^Department of Internal Medicine, Kaohsiung Municipal Siaogang Hospital, Kaohsiung Medical University Hospital, Kaohsiung Medical University, Kaohsiung, Taiwan; ^5^Division of Nephrology, Department of Internal Medicine, Kaohsiung Medical University Hospital, Kaohsiung Medical University, Kaohsiung, Taiwan; ^6^Faculty of Medicine, College of Medicine, Kaohsiung Medical University, Kaohsiung, Taiwan; ^7^Division of Gastroenterology, Department of Internal Medicine, Kaohsiung Medical University Hospital, Kaohsiung Medical University, Kaohsiung, Taiwan; ^8^Research Center for Precision Environmental Medicine, Kaohsiung Medical University, Kaohsiung, Taiwan

**Keywords:** secondhand smoke, peptic ulcer disease, gastroesophageal reflux disease, Taiwan Biobank, risk factors

## Abstract

**Background:**

Active smokers are known to be at an increased risk of both gastroesophageal reflux disease (GERD) and peptic ulcer disease (PUD), however the role of passive smoking remains unclear. In this study, we aimed to examine whether secondhand smoke (SHS) is associated with PUD and GERD.

**Methods:**

In this population-based study, we conducted a large-scale analysis with 88,297 never-smokers (male: 18,595; female: 69,702; mean age 50.1 ± 11.0 years) from the Taiwan Biobank. The exposure group was comprised of those who had been exposed to SHS, and the no exposure group as those without SHS exposure. According to the frequency of exposure, we further divided the participants into “no exposure,” “<1 h per week,” and “≥1 h per week” groups. A cutoff point of 1 h per week was chosen according to the median exposure time in our participants. Associations between SHS and SHS frequency with PUD and GERD were assessed.

**Results:**

Of the 88,297 enrolled participants, 11,909 (13.5%) had PUD and 76,388 (86.5%) did not. In addition, 11,758 (13.3%) had GERD and 76,539 (86.7%) did not. Multivariable analysis showed a significant association between SHS with PUD (odds ratio [OR] = 1.166; 95% confidence interval [CI] = 1.084–1.254; *p* < 0.001), and GERD (OR = 1.131; 95% CI = 1.053–1.216; *p* = 0.001). Furthermore, those exposed to SHS ≥ 1 h per week (vs. no exposure) were associated with higher risks of PUD (OR = 1.232; 95% CI = 1.121–1.355; *p* < 0.001) and GERD (OR = 1.200; 95% CI = 1.093–1.319; *p* < 0.001).

**Conclusion:**

SHS was significantly associated with PUD and GERD. Furthermore, exposure to SHS ≥ 1 h per week (vs. no exposure) was associated with a 1.23-fold higher risk of PUD and 1.20-fold higher risk of GERD. This study represents the largest population-based investigation to explore the association between SHS with PUD and GERD in Taiwanese never-smokers.

## Introduction

Peptic ulcer disease (PUD) is a gastrointestinal mucosal defect, characterized by asymptomatic or symptomatic abdominal pain, bloating, and abdominal fullness (1). The Global Burden of Disease, Injuries and Risk Factors Study reported that approximately 8 million people had PUD in 2019 worldwide (2). In addition, in Taiwan, a prospective study of 6,457 individuals undergoing health examinations reported that of those diagnosed with PUD in esophagogastroduodenoscopy, two-thirds were asymptomatic (3). Proposed risk factors for PUD include increased body mass index (BMI), smoking tobacco, malignancy, stress, radiation therapy, non-steroidal anti-inflammatory drugs, chemotherapy, and *helicobacter pylori* infection (3–5). Complications include ulcer bleeding, gastric outlet obstruction, bowel penetration and fistulation, and even perforation.

The 2006 Montreal Definition and Classification defined gastroesophageal reflux disease (GERD) as troublesome symptoms and/or complications caused by reflux of stomach contents (6). The estimated incidence of GERD is 5.0 per 1,000 person-years globally, with a lower prevalence in Asia than in Western countries (7). In addition, the reported weekly prevalence of gastroesophageal reflux symptoms is highest in Southeast Europe and South Asia (>25%) followed by Central America (19.6%), and lowest in Southeast Asia (7.4%) (7–9). A recent meta-analysis concluded that increased BMI, tobacco smoking, heredity, and *Helicobacter pylori* infection were the major risk factors for GERD (9, 10). Other risk factors including alcohol consumption and dietary factors (10) have also been proposed. Established complications include esophageal adenocarcinoma, Barrett’s esophagus, reflux esophagitis, and reflux stricture, while proposed complications include idiopathic pulmonary fibrosis and recurrent otitis media (6). Due to the growing incidence of PUD and GERD and their complications (2, 8), more research is needed to elucidate the associated risk factors.

Smoking is associated with increased prevalence and mortality of many diseases, including atherosclerotic cardiovascular disease, malignancy, chronic obstructive pulmonary disease, infection, osteoporosis, hip fracture, reproductive disorders, PUD, periodontal disease and ophthalmologic disorders (11–17). Notably, many studies have also reported the deleterious effects of secondhand smoke (SHS). According to the 2012 International Agency for Research on Cancer published report, SHS mainly consisted of two parts. The first part compromised burning end of a cigarette (or other burned tobacco compounds), which called diluted sidestream smoke. The second part includes smoke emitted during puffing and gasses diffused during smoking through the cigarette paper, which also called exhaled mainstream smoke (18). SHS was also names as passive smoking, which signified its involuntariness (18). A 2011 retrospective study (19) with data from 192 countries identified associations between SHS exposure with asthma, premature death, lower respiratory infection, and ischemic heart disease. SHS has also been associated with an elevated risk of lung cancer (20), cardiovascular disease (21–23), stroke (11, 24), type 2 diabetes mellitus (DM) (25), gestational DM (26, 27), and female sexual dysfunction (28). Regarding gastrointestinal diseases, a 2021 Japanese case–control study disclosed that people exposed to passive smoking at home had a higher risk of developing ulcerative colitis (29). In addition, a study (30) of people who had never used tobacco reported that higher SHS exposure was correlated with an increased risk of esophageal squamous cell carcinoma. Moreover, a case–control study (31) of children with pathologically confirmed esophagitis reported a significantly higher risk of esophagitis in those whose parents smoked, and a retrospective study (32) of 34 infants reported that those exposed to environmental tobacco smoke (ETS) had elevated pH parameters and higher reflux index. Furthermore, the authors emphasized that ETS was a strong risk factor for infantile gastroesophageal reflux (32).

Despite these findings, few studies have investigated the associations between SHS with PUD and GERD. Therefore, the aims of this population-based study was to examine the association between SHS with PUD and GERD in a large cohort of never-smokers from the Taiwan Biobank (TWB), and also to determine the relative risk of SHS exposure frequency with PUD and GERD.

## Materials and methods

### TWB

The TWB is a pioneering project initiated in 2008 with the aim of advancing healthcare in Taiwan by recording health information including genetic and lifestyle factors and storing biological samples from Taiwanese participants (33, 34). Ethical approval for the TWB was granted by the Institutional Review Board on Biomedical Science Research, Academia Sinica, Taiwan and the Ethics and Governance Council of the TWB.

### Ethics statement

All enrollees in the TWB are requested to sign written informed consent forms. The current study was approved by the Institutional Review Board of Kaohsiung Medical University Hospital (KMUHIRB-E(I)-20210058), and was conducted following the Helsinki Declaration.

### Study participants

**Figure 1:**
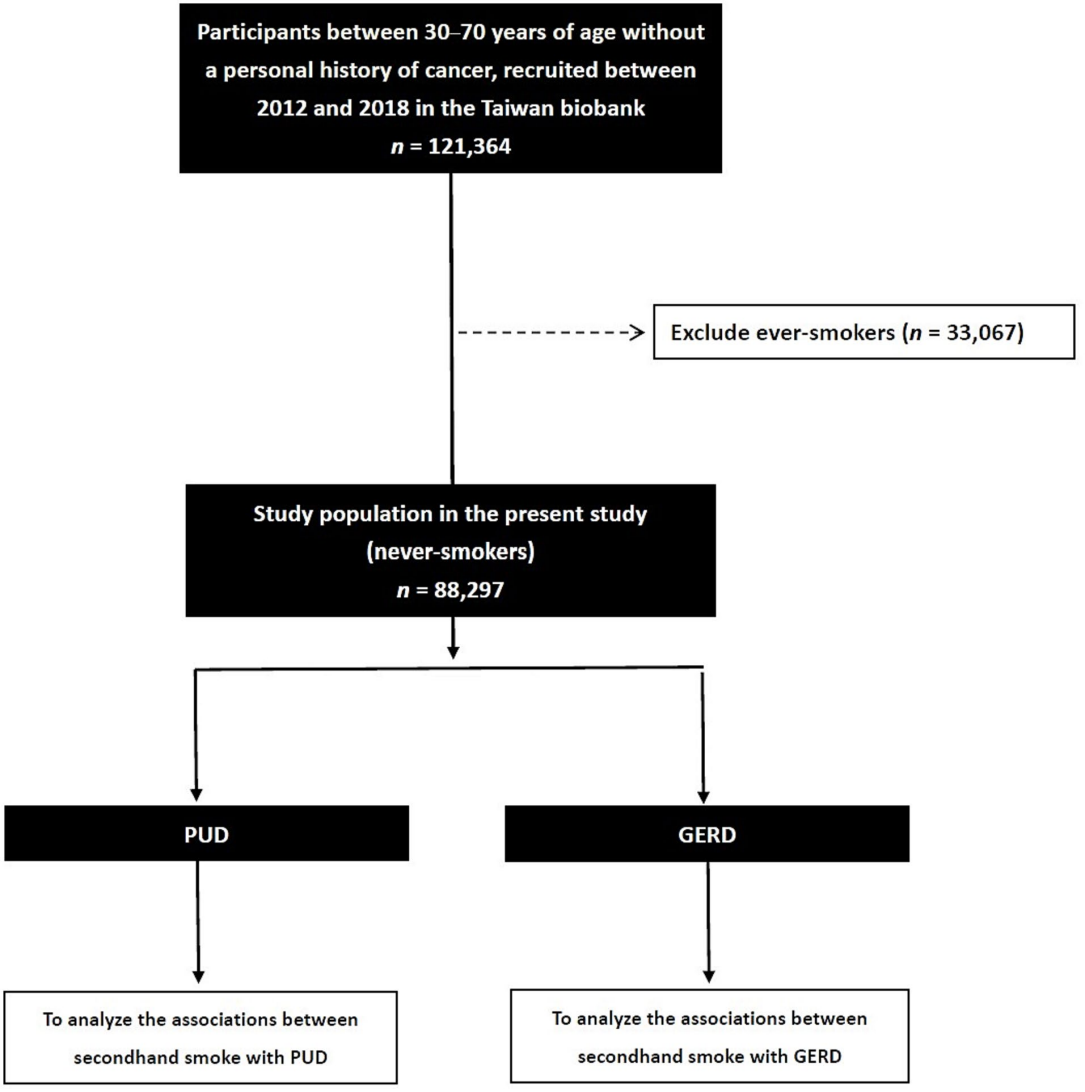
Flowchart of study population.

Of the 121,364 subjects enrolled in the TWB, those with a history of smoking (*n =* 33,067) were excluded from this study, and the remaining 88,297 were included (male: 18,595; female: 69,702; mean age 50.1 ± 11.0 years) for analysis (Figure 1).

### Collection of study variables

We recorded body height and weight, and systolic and diastolic blood pressures (BP). The average of three BP measurements taken after a 1–2-min break using an electronic BP device after refraining from smoking, exercise and caffeine intake for at least 30 min was used in the analysis. In addition, information on the presence of hypertension and DM, smoking status, age and sex were also obtained, and the participants were also asked whether they had a history of PUD or GERD. Those who reported a history were then classified into the PUD or GERD group accordingly.

Other variables of interest included estimated glomerular filtration rate [eGFR; calculated as reported previously (35)], triglycerides, total cholesterol, high- and low-density lipoprotein cholesterol (HDL-C/LDL-C), hemoglobin, uric acid and fasting glucose.

### Smoking and SHS assessments

We first grouped the participants as “never-smokers,” “ex-smokers,” or “active smokers” according to questionnaires which they were asked to complete. The never-smokers were then asked whether they had ever been exposed to SHS. Those who replied that they had were classified into the exposure group, and those without exposure to SHS were classified into the no exposure group. The participants who had been exposed to SHS were then asked, “How many hours per week have you been exposed to SHS?” According to their answer, they were further classified into “no exposure,” “<1 h per week,” and “≥1 h per week” groups. The cutoff point of 1 h per week was chosen according to the median time of exposure in our participants.

### Statistical analysis

The study variables are presented in percentage or mean ± SD as appropriate. Independent t-tests were used for comparing differences in continuous variables, and chi-square tests were used for categorical variables. Factors associated with PUD and/or GERD were identified by multivariable logistic regression. All tests were two-tailed, and a statistically significant association was considered at a *p-*value <0.05. SPSS was used for the analysis (v19, IBM Inc., Armonk, NY, United States).

## Results

The 88,297 participants were classified into those with PUD (*n* = 11,909; 13.5%) or without PUD (*n* = 76,388; 86.5%), and with GERD (*n* = 11,758; 13.3%) or without GERD (*n* = 76,539; 86.7%).

### Clinical characteristics of the PUD groups

The clinical characteristics of the participants with and without PUD are shown in Table 1. Compared to the participants without PUD, those with PUD were older, predominantly male, had higher prevalence rates of DM and hypertension, higher prevalence rates of alcohol and betel nut chewing history, higher prevalence of regular exercise habit, higher systolic BP, higher fasting glucose, higher hemoglobin, higher triglyceride, higher total cholesterol, higher LDL-C and lower eGFR.

**Table 1:** Clinical characteristics of the study participants classified by the presence of PUD.

Characteristics	PUD (*n* = 88,297)		
	PUD (−) (*n* = 76,388)	PUD (+) (*n* = 11,909)	*p*
Age (year)	49.5 ± 11.1	53.5 ± 10.0	<0.001
Male sex (%)	20.8	22.5	<0.001
DM (%)	4.3	6.1	<0.001
Hypertension (%)	10.4	13.9	<0.001
Secondhand smoke (%)	7.9	8.1	0.527
Alcohol history (%)	2.9	3.3	0.039
Betel nut chewing history (%)	0.48	0.64	0.021
Regular exercise habits (%)	40.6	45.7	<0.001
Systolic BP (mmHg)	119.2 ± 18.8	119.9 ± 18.2	<0.001
Diastolic BP (mmHg)	72.7 ± 11.2	72.6 ± 10.8	0.154
Body mass index (kg/m^2^)	23.9 ± 3.7	23.7 ± 3.6	<0.001
Laboratory parameters			
Fasting glucose (mg/dL)	94.8 ± 19.4	95.8 ± 18.1	<0.001
Hemoglobin (g/dL)	13.4 ± 1.5	13.5 ± 1.5	<0.001
Triglyceride (mg/dL)	107.4 ± 78.2	109.7 ± 80.3	0.003
Total cholesterol (mg/dL)	196.3 ± 35.8	198.0 ± 35.5	<0.001
HDL-C (mg/dL)	56.3 ± 13.3	56.5 ± 13.7	0.068
LDL-C (mg/dL)	120.7 ± 31.7	121.6 ± 31.3	0.004
eGFR (mL/min/1.73 m^2^)	105.8 ± 24.1	102.7 ± 23.9	<0.001
Uric acid (mg/dL)	5.2 ± 1.3	5.2 ± 1.3	0.959

PUD, peptic ulcer disease; DM, diabetes mellitus; BP, blood pressure; HDL-C, high-density lipoprotein cholesterol; LDL-C, low-density lipoprotein cholesterol; eGFR, estimated glomerular filtration rate; BMI, body mass index.

### Association of SHS with PUD

Multivariable analysis adjusting for age, sex, hypertension, DM, SHS, alcohol intake, betel quid chewing, regular exercise, systolic BP, BMI, hemoglobin, fasting glucose, triglycerides, total cholesterol, LDL-C and eGFR, showed significant associations between SHS (odds ratio [OR] = 1.166; 95% confidence interval [CI] = 1.084–1.254), old age, male sex, hypertension, DM, low systolic BP, low BMI (all *p* < 0.001), no regular exercise and low fasting glucose (both *p* = 0.003), and high hemoglobin (*p* = 0.014) with PUD (Table 2).

**Table 2:** Association of secondhand smoke with PUD using multivariable logistic regression analysis in never smokers (*n* = 88,297).

Variables	Multivariable (PUD)	
	Odds ratio (95% CI)	*p*
Age (per 1 year)	1.040 (1.038–1.043)	<0.001
Male vs. female	1.175 (1.106–1.248)	<0.001
DM	1.184 (1.075–1.304)	0.001
Hypertension	1.163 (1.091–1.239)	<0.001
Secondhand smoke	1.166 (1.084–1.254)	<0.001
Alcohol history	1.050 (0.938–1.176)	0.395
Betel nut chewing history	1.179 (0.914–1.520)	0.206
Regular exercise habits	0.939 (0.901–0.980)	0.003
Systolic BP (per 1 mmHg)	0.993 (0.991–0.994)	<0.001
Body mass index (per 1 kg/m^2^)	0.982 (0.975–0.988)	<0.001
Fasting glucose (per 1 mg/dL)	0.998 (0.997–0.999)	0.003
Hemoglobin (per 1 g/dL)	1.021 (1.004–1.038)	0.014
Triglyceride (per 1 mg/dL)	1.000 (1.000–1.000)	0.150
Total cholesterol (per 1 mg/dL)	0.999 (0.998–1.001)	0.235
LDL-C (per 1 mg/dL)	1.001 (0.999–1.002)	0.403
eGFR (per 1 mL/min/1.73 m^2^)	0.999 (0.998–1.000)	0.164

Values expressed as odds ratio and 95% confidence interval (CI). Abbreviations are the same as in Table 1. Adjusted for age, sex, DM and hypertension, secondhand smoke, alcohol and betel nut chewing history, regular exercise habit, systolic BP, BMI, fasting glucose, hemoglobin, triglyceride, total cholesterol, LDL-cholesterol and eGFR.

### Clinical characteristics of the GERD groups

The clinical characteristics of the participants with and without GERD are shown in Table 3. Compared to the participants without GERD, those with GERD were older, predominantly female, had higher prevalence rates of DM and hypertension, higher prevalence rates of alcohol history, higher prevalence of regular exercise habit, higher fasting glucose, higher triglyceride, higher total cholesterol, higher LDL-C, lower eGFR and lower uric acid.

**Table 3:** Clinical characteristics of the study participants classified by the presence of GERD.

Characteristics	GERD (*n* = 88,297)		
	GERD (−) (*n* = 76,539)	GERD (+) (*n* = 11,758)	*p*
Age (year)	49.7 ± 11.1	52.5 ± 10.3	<0.001
Male sex (%)	21.5	18.3	<0.001
DM (%)	4.4	5.8	<0.001
Hypertension (%)	10.4	13.9	<0.001
Secondhand smoke (%)	7.9	8.2	0.267
Alcohol history (%)	2.9	3.3	0.010
Betel nut chewing history (%)	0.50	0.51	0.858
Regular exercise habits (%)	40.8	44.2	<0.001
Systolic BP (mmHg)	119.3 ± 18.8	119.6 ± 17.8	0.066
Diastolic BP (mmHg)	72.7 ± 11.2	72.6 ± 10.6	0.202
Body mass index (kg/m^2^)	23.9 ± 3.7	23.9 ± 3.7	0.593
Laboratory parameters			
Fasting glucose (mg/dL)	94.8 ± 19.3	95.5 ± 18.2	<0.001
Hemoglobin (g/dL)	13.4 ± 1.5	13.4 ± 1.4	0.322
Triglyceride (mg/dL)	107.2 ± 77.9	111.0 ± 82.3	<0.001
Total cholesterol (mg/dL)	196.2 ± 35.9	198.6 ± 35.0	<0.001
HDL-C (mg/dL)	56.3 ± 13.4	56.4 ± 13.5	0.315
LDL-C (mg/dL)	120.7 ± 31.8	121.9 ± 31.1	<0.001
eGFR (mL/min/1.73 m^2^)	105.6 ± 24.1	104.0 ± 23.9	<0.001
Uric acid (mg/dL)	5.2 ± 1.3	5.2 ± 1.3	0.007

GERD, gastroesophageal reflux disease. Other abbreviations are the same as in Table 1.

### Association of SHS with GERD

Multivariable analysis adjusting for age, sex, hypertension, DM, SHS, alcohol intake, regular exercise, uric acid, fasting glucose, triglycerides, total cholesterol, eGFR and LDL-cholesterol showed significant associations between SHS (OR = 1.131; 95% CI = 1.053–1.216), alcohol history, low fasting glucose (all *p* = 0.001), old age, female sex, low uric acid, hypertension (all *p* < 0.001), DM (*p* = 0.003), and high triglycerides (*p* = 0.005) with GERD (Table 4).

**Table 4:** Association of secondhand smoke with GERD using multivariable logistic regression analysis in never smokers (*n* = 88,297).

Variables	Multivariable (GERD)	
	Odds ratio (95% CI)	*p*
Age (per 1 year)	1.023 (1.021–1.025)	<0.001
Male vs. female	0.865 (0.816–0.916)	<0.001
DM	1.164 (1.055–1.284)	0.003
Hypertension	1.158 (1.089–1.232)	<0.001
Secondhand smoke	1.131 (1.053–1.216)	0.001
Alcohol history	1.204 (1.077–1.346)	0.001
Regular exercise habits	0.969 (0.929–1.011)	0.143
Fasting glucose (per 1 mg/dL)	0.998 (0.997–0.999)	0.001
Triglyceride (per 1 mg/dL)	1.000 (1.000–1.001)	0.005
Total cholesterol (per 1 mg/dL)	1.000 (0.999–1.002)	0.606
LDL-C (per 1 mg/dL)	1.000 (0.999–1.001)	0.924
eGFR (per 1 mL/min/1.73 m^2^)	0.999 (0.998–1.000)	0.199
Uric acid (per 1 mg/dL)	0.966 (0.948–0.984)	<0.001

Values expressed as odds ratio and 95% confidence interval (CI). Abbreviations are the same as in Table 1. Adjusted for age, sex, DM and hypertension, secondhand smoke and alcohol history, regular exercise habit, fasting glucose, triglyceride, total cholesterol, LDL-cholesterol, eGFR and uric acid.

### Association between SHS frequency with PUD and GERD

After adjusting for confounders, the participants who were exposed to SHS ≥ 1 h per week (vs. no exposure; OR = 1.232; 95% CI = 1.121–1.355; *p* < 0.001) were associated with a higher risk of PUD. In addition, those who were exposed to SHS ≥ 1 h per week (vs. no exposure; OR = 1.200; 95% CI = 1.093–1.319; *p* < 0.001) were associated with a higher risk of GERD (Table 5).

**Table 5:** Relative risk for PUD and GERD according to frequency of secondhand smoke in never smokers (*n* = 88,297).

Variables	Multivariable	
	Odds ratio (95% CI)	*p*
PUD^a^		
No exposure	Reference	
<1 h per week exposure	1.111 (0.992–1.245)	0.069
≥1 h per week exposure	1.232 (1.121–1.355)	<0.001
GERD^b^		
No exposure	Reference	
<1 h per week exposure	1.067 (0.953–1.195)	0.260
≥1 h per week exposure	1.200 (1.093–1.319)	<0.001

Values expressed as odds ratio and 95% confidence interval (CI). Abbreviations are the same as in Table 1.

a Adjusted for age, sex, DM and hypertension, secondhand smoke, alcohol and betel nut chewing history, regular exercise habit, systolic BP, BMI, fasting glucose, hemoglobin, triglyceride, total cholesterol, LDL-cholesterol and eGFR.

b Adjusted for age, sex, DM and hypertension, secondhand smoke and alcohol history, regular exercise habit, fasting glucose, triglyceride, total cholesterol, LDL-cholesterol, eGFR and uric acid.

### Association of smoke status with PUD using multivariable logistic regression analysis

Supplementary Table S1 showed association of smoke status with PUD using multivariable logistic regression analysis in all participants (*n* = 121,364). Multivariable analysis showed that never smokers, SHS (+) [vs. never smokers, SHS (−); OR = 1.163; 95% CI = 1.082–1.249; *p* < 0.001], and ex-, or active smokers [vs. never smokers, SHS (−); OR = 1.274; 95% CI = 1.218–1.331; *p* < 0.001] were significantly associated with PUD.

### Association of smoke status with GERD using multivariable logistic regression analysis

Supplementary Table S2 showed association of smoke status with GERD using multivariable logistic regression analysis in all participants (*n* = 121,364). Multivariable analysis showed that never smokers, SHS (+) [vs. never smokers, SHS (−); OR = 1.124; 95% CI = 1.046–1.207; *p* < 0.001], and ex-, or active smokers [vs. never smokers, SHS (−); OR = 1.264; 95% CI = 1.209–1.322; *p* < 0.001] were significantly associated with GERD.

## Discussion

The results of this population-based study of 88,297 participants showed that the individuals in the SHS exposure group had increased risks of PUD and GERD. Furthermore, compared with no exposure, the participants exposed to SHS for ≥1 h per week were associated with a 1.23-fold higher risk of PUD and 1.20-fold higher risk of GERD. Furthermore, ex-, or active smokers also had increased risks of PUD and GERD.

The first important finding of this study is the association between SHS and PUD. A previous rat study (36) designed a 20-L smoke chamber with smoke/air mixture continuously delivered at a flow rate of 250 mL/h for a 1-h experimental period, and then applied an oral dose of 70% ethanol in a volume of 10 mL/kg 15 min later. The results showed that a higher smoke concentration resulted in larger ethanol-induced gastric mucosal lesions. However, there were no significant differences in possible stress factors including serum pH, partial pressure of carbon dioxide, partial pressure of oxygen, bicarbonate, systemic BP or heart rate before and after tobacco and alcohol exposure. Active smoking was simulated using nicotine administered via an oral route, but this study is the first and to date the only paper to demonstrate that passive smoking with nicotine absorbed through the respiratory tract also worsened ethanol-induced gastric ulcers in a rat model. Although very few studies have elucidated the pathogenesis between SHS and PUD, an increasing amount of research has been conducted to explain the relationship between active smoking and PUD. Ulcer healing (37) involves ulcer margin epithelial cell proliferation, migration, angiogenesis, gastric gland reconstruction and migration. Previous studies (38–40) have demonstrated the positive effects of nitric oxide (NO), prostaglandin E2, and vascular endothelial growth factor on vasodilation, increased mucosal blood flow, and angiogenesis. In another experimental rat model (41) of acetic acid-induced ulcers, rats were exposed to 0, 2% or 4% concentrations of tobacco smoke for 1 h daily for 6 days. The ulcers healed spontaneously after 10 days in the control group, but delayed ulcer healing was noted in the smoke-exposed group (*p* < 0.05 in the 2% group and *p* < 0.01 in the 4% group). In subgroup analysis (41), exposure to tobacco smoke at 4% concentration was associated with a marked reduction in ulcer base constitutive NO synthase activity and ulcer margin micro-vessels. In addition, caspase-3 was found to be activated during apoptosis during the process of programmed cell death. In another rodent study (42), the smoke-exposed group showed higher levels of activated caspase-3 in immunohistochemistry compared with the air-exposed group. Epidermal growth factor (EGF) plays a vital role in gastrointestinal ulcer cell reconstruction and proliferation (43), and it is mainly formed by the salivary glands, Brunner’s glands in the duodenum, and pancreas (44). Konturek et al. (44) recruited 36 healthy male volunteers and asked them to smoke one cigarette every 30 min. EGF concentrations were then measured and found to be significantly decreased in the saliva and duodenum. Ma et al. (45) also reported decreased serum and gastric mucosal EGF concentrations as well as salivary gland EGF messenger ribonucleic acid expression in the smoke-exposed group of acetic acid-induced ulcer rats. Surprisingly, the intravenous administration of EGF reduced ulcer size, angiogenesis, and muscular cell proliferation in the smoke-exposed group (45). In summary, active smoking affects ulcer site healing by increasing apoptosis, inhibiting angiogenesis, and decreasing vasodilation and reconstruction based on current evidence. The aforementioned mechanisms may partially explain the association between SHS and PUD.

Our results also showed an association between SHS and GERD. One case–control study (31) of 278 children with esophageal biopsy-confirmed esophagitis identified a 6-fold higher risk of esophagitis if at least one parent smoked (*p* < 0.001; relative risk 6.1, 95% CI 3.2 to 11.3). Proposed pathogeneses include a combination of increased free radical producing activity and a lower antioxidant level (31, 46). Monajemzadeh et al. (47) investigated the association between nicotinine, a nicotine metabolite, and esophagitis in children with GERD, and found an increased risk of developing esophagitis in children exposed to ETS. However, there is currently little evidence to explain the association between SHS exposure and GERD in adults. Several studies have investigated the connection between active smoking and GERD. One study (48) using 24-h ambulatory esophageal pH monitoring showed that chronic smokers had more reflux episodes and increased duration of esophageal acid exposure. Many studies (48–50) have found that decreased gastroesophageal sphincter pressure contributes to GERD in smokers. Dennish and Castell (49) enrolled six normal men who were chronic smokers and studied the relationship between smoking and lower esophageal sphincter pressure using a triple-lumen polyvinyl tube. They found that the mean lower esophageal sphincter pressure fell to 11.4 mmHg from a baseline value of 19.6 mmHg 2 to 3 min after smoking, and the difference was significant (*p* < 0.001). Moreover, they found that the gastroesophageal sphincter pressure returned to baseline level 5 min after the men stopped smoking. Stanciu and Bennett (48) also found a significant change in end-expiratory gastroesophageal sphincter pressure before and after smoking (from a mean 10.8 cmH_2_O to 6.4 cmH_2_O, *p* < 0.01). Stanciu and Bennett (48) hypothesized that this decrease may be due to the cholinergic system being blocked by nicotine, as a previous *in-vitro* study found that nicotine could cause relaxation of lower esophagus circular muscle fibers. Kahrilas and Gupta (51) further confirmed these findings, as they showed that smokers with heartburn and endoscopically confirmed esophagitis had a lower esophagus sphincter pressure compared with asymptomatic smokers. Smoking has also been shown to inhibit acid-clearing capacity as assessed using a modified acid-clearing test (52). Another study Koelz et al. (53) also found that smoking was positively correlated with ranitidine treatment failure in patients with peptic esophagitis during a 6-week treatment period. Taken together, these studies shed light on the possible pathophysiology of active smoking and GERD, including more reflux episodes, increased duration of exposure to esophageal acid, reduced acid-clearing ability, and decreased lower gastroesophageal sphincter pressure.

In the study, multivariable analysis (Table 2) showed that high hemoglobin was associated with a high risk of PUD, and regular exercise habit was associated with a low risk of PUD. Stress-induced hemoconcentration (54) may be the reasons of high hemoglobin in PUD group. Hemoconcentration attributes to the conditions when the ratio of serum cellular components to the plasma volume increases, especially red blood cells (55). Stress-hemoconcentration specified the condition that an acute elevation of blood pressure and a net efflux of plasma into third spacing which results in increased colloid osmotic pressure and raised plasma protein concentration after stressors (55). Evidence (56) has shown that the presence of gastro-intestinal ulcer after burns is directly connected to the occurrence of hemoconcentration. As for the association between regular exercise habit and PUD, there is growing evidence that the circulation cells of the innate immune system and the anti-inflammatory and antioxidant effect increase after exercise (57). In one review article, there was evidence that increased physical activity enhance the ability to deal with physical distress and anxiety (58). In the experimental study, peak gastric acid level had fallen to about 60% after exercise with statistically significance (59). Study had shown reduced risk of duodenal ulcer formation in physical active individuals (60). Decrease gastric acid secretion, rebust innate immune system, increase anti-inflammatory effect may explain regular exercise habit with low risk of PUD.

Various social, psychological, and biological factors also play a role in the development of PUD. In some population-based studies, stress, depressed mood (61), suicidal thoughts (61), panic disorders (62), and childhood abuse (63) showed positively correlation with PUD. However, TWB did not collect psychological status of the involved volunteers and thus may underestimated their effect on occurrence of PUD.

The results of this study are enhanced by the inclusion of a large cohort. The limitations of this cross-sectional study include that the durations of PUD and GERD were not evaluated, so that causal relationships between SHS with PUD and GERD could also not be evaluated. Longitudinal studies are needed to investigate this issue. Another limitation is that the occurrence of PUD and GERD was ascertained through the participants’ responses to questionnaires without endoscopic verification, and thus their type and severity could not be ascertained, which may lead to incorrect information due to recall bias. However, a previous study (64) from Taiwan noted moderate agreement between claims records and diseases identified through questionnaires. In addition, we did not conduct subgroup analyses on the brand of cigarette, the extent and amount of SHS, and the place and distance where the non-smoker was exposed. Fourth, the TWB collects health-related data on healthy volunteers across Taiwan, and women may be more willing or able to participate in research studies compared with men due to greater health awareness. Thus, our findings may not be generalizable to the general population. Finally, the generalizability of our findings may be limited by the ethnicity of our participants, all of whom were of Chinese ethnicity.

In conclusion, we found significant associations between SHS with PUD and GERD. Furthermore, exposure to SHS for ≥1 h per week (vs. no exposure) was associated with 1.23- and 1.20-fold higher risks of PUD and GERD, respectively. This study represents the largest population-based investigation to date to explore the association between SHS with PUD and GERD in Taiwanese never-smokers.

## Data Availability

The raw data supporting the conclusions of this article will be made available by the authors, without undue reservation.
