# Supplementary material for: Secondhand smoke is associated with peptic ulcer disease and gastroesophageal reflux disease in non-smokers in a large Taiwanese population study
Source: Front Public Health. 2024 Oct 7;12:1450481. doi: 10.3389/fpubh.2024.1450481 (PMC11491381; doi:10.3389/fpubh.2024.1450481)
Supplement: Supplementary file 1 [file Data_Sheet_1.docx]

Supplemental table 1. Association of smoke status with PUD using multivariable logistic regression analysis in all participants (*n* = 121,364)

| Variables | Multivariable (PUD) | |
| --- | --- | --- |
|  | Odds ratio (95% CI) | *p* |
| Age (per 1 year) | 1.038 (1.036-1.040) | < 0.001 |
| Male *vs.* female | 1.123 (1.068-1.181) | < 0.001 |
| DM | 1.142 (1.057-1.233) | 0.001 |
| Hypertension | 1.152 (1.096-1.212) | < 0.001 |
| Smoke status |  |  |
| Never smokers, SHS (-) | Reference |  |
| Never smokers, SHS (+) | 1.163 (1.082-1.249) | < 0.001 |
| Ex-, or active smokers | 1.274 (1.218-1.331) | < 0.001 |
| Alcohol history | 1.067 (1.005-1.132) | 0.033 |
| Betel nut chewing history | 1.198 (1.118-1.282) | < 0.001 |
| Regular exercise habits | 0.942 (0.910-0.975) | 0.001 |
| Systolic BP (per 1 mmHg) | 0.993 (0.992-0.994) | < 0.001 |
| Body mass index (per 1 kg/m^2^) | 0.980 (0.975-0.985) | < 0.001 |
| Fasting glucose (per 1 mg/dL) | 0.998 (0.997-0.999) | < 0.001 |
| Hemoglobin (per 1 g/dL) | 1.016 (1.002-1.030) | 0.024 |
| Triglyceride (per 1 mg/dL) |  |  |
| Total cholesterol (per 1 mg/dL) |  |  |
| LDL-C (per 1 mg/dL) |  |  |
| eGFR (per 1 mL/min/1.73 m^2^) |  |  |

Values expressed as odds ratio and 95% confidence interval (CI). Abbreviations are the same as in Table 1.

Adjusted for age, sex, DM and hypertension, smoke status, alcohol and betel nut chewing history, regular exercise habit, systolic BP, BMI, fasting glucose, hemoglobin, triglyceride, total cholesterol, LDL-cholesterol and eGFR.

Supplemental table 2. Association of smoke status with GERD using multivariable logistic regression analysis in all participants (*n* = 121,364)

| Variables | Multivariable (GERD) | |
| --- | --- | --- |
|  | Odds ratio (95% CI) | *p* |
| Age (per 1 year) | 1.019 (1.017-1.021) | < 0.001 |
| Male *vs.* female | 0.819 (0.781-0.858) | < 0.001 |
| DM | 1.152 (1.064-1.248) | < 0.001 |
| Hypertension | 1.175 (1.118-1.235) | < 0.001 |
| Smoke status |  |  |
| Never smokers, SHS (-) | Reference |  |
| Never smokers, SHS (+) | 1.124 (1.046-1.207) | 0.001 |
| Ex-, or active smokers | 1.264 (1.209-1.322) | < 0.001 |
| Alcohol history | 1.165 (1.096-1.237) | < 0.001 |
| Regular exercise habits | 0.982 (0.947-1.017) | 0.303 |
| Fasting glucose (per 1 mg/dL) | 0.998 (0.997-0.999) | < 0.001 |
| Triglyceride (per 1 mg/dL) | 1.000 (1.000-1.001) | 0.003 |
| Total cholesterol (per 1 mg/dL) | 1.001 (1.000-1.002) | 0.235 |
| LDL-C (per 1 mg/dL) | 1.000 (0.999-1.001) | 0.985 |
| eGFR (per 1 mL/min/1.73 m^2^) | 0.999 (0.999-1.000) | 0.107 |
| Uric acid (per 1 mg/dL) | 0.965 (0.951-0.979) | < 0.001 |

Values expressed as odds ratio and 95% confidence interval (CI). Abbreviations are the same as in Table 1.

Adjusted for age, sex, DM and hypertension, smoke status and alcohol history, regular exercise habit, fasting glucose, triglyceride, total cholesterol, LDL-cholesterol, eGFR and uric acid.
